# What alternative and innovative domestic methods of healthcare financing can be explored to fix the current claims reimbursement challenges by the National Health Insurance Scheme of Ghana? Perspectives of health managers

**DOI:** 10.1186/s12962-021-00323-2

**Published:** 2021-10-09

**Authors:** Alexander Suuk Laar, Michael Asare, Philip Ayizem Dalinjong

**Affiliations:** 1 REJ Institute, Research and ICT Consultancy Services, Post Office Box SN 336, Tamale, Ghana; 2 Holy Family Hospital, Nkawkaw, Ghana; 3 Navrongo Health Research Centre, Navrongo, Ghana

**Keywords:** Universal Health Coverage, National Health Insurance Scheme, Alternative local funding, Health managers, Healthcare financing challenges, Sustainability, Ghana

## Abstract

**Background:**

Low-and-middle -income countries (LMICs), to achieve sustainable universal health coverage (UHC) governments are implementing local and sustainable methods of healthcare financing. However, in Ghana, there is limited evidence on these local methods for healthcare financing to inform policy. This study aimed at exploring health managers views on alternative domestic and sustainable methods of healthcare financing for UHC under the National Health Insurance Scheme (NHIS).

**Methods:**

A qualitative study using in-depth interviews with 16 health facility managers were held. The health facilities and participants were selected using convenience and purposive sampling methods. A written consent was obtained from participants prior to participation in the interview. Data was transcribed verbatim and analyzed using thematic framework approach.

**Results:**

Health managers across all the health facilities mentioned delayed and erratic claims reimbursement to health facilities as the main challenge. Participants attributed the main reason to lack of funds by the National Health Insurance Authority (NHIA). They said the delayed and irregular payments has been a challenge to efficient delivery of quality healthcare to clients. That in some instances they have been compelled to demand cash or out-of-pocket payment from insured clients or insurance card bearers to be able to render needed healthcare services to them. Participants think that to ensure regular reimbursement of claims to the health facilities by the NHIA, the managers think alternative local sources of funding need to be explored to fill the funding gap. To put in place this, they suggested the need to start levying special taxes on natural resources such as crude oil and gas, gold, bauxite, cocoa, mobile money transfers, airtime and increasing the proportion of levies on the existing Value Added Tax (VAT).

**Conclusion:**

The study provides important insights into potential innovative alternative domestic sources for raising additional funds to finance healthcare services in Ghana. Despite the potential of these sources, it is important for governments and health policy makers in Ghana and other LMICs who are working towards implementing innovative local methods using special levies on mobile communication services and natural resources to finance their UHC, to implement those that best suit their economies to ensure equity for better health.

## Background

Sustainable healthcare financing is one of the key components for the achievement of universal health coverage (UHC [1]). UHC is defined as providing financial protection from the costs of using health services for all people of a country, as well as enabling them to obtain the health services that they need, where these services should be of sufficient quality to be effective [2]. Emphasis has been placed on how to finance health systems to move towards UHC [3–5]. Financing UHC is complex and difficult in Low -and -Middle -Income Countries (LMICs) especially where the informal sector dominates [6, 7]. Health care financing continues to generate heated discussion in LMICs, especially how this could be sustainably carried out [8]. Health policy makers and other stakeholders are particularly challenged in this direction [8, 9]. Universal access to healthcare and sustainable financing are important health system goals [10], since sound financing strategies tend to impact on access to health services and health outcomes [11]. However, adequate and sustainable financing of the public health system continues to evade most LMICs, in which health systems struggle with scant and inequitably distributed resources [11].

As part of renewed efforts at pursuing sustainable health care financing options that promote UHC, Ghana has adopted a National Health Insurance Scheme (NHIS) to provide universal access to healthcare for all at an affordable cost [12–16]. The sources of the Scheme’s funds come from members registration fees and an indirect tax (VAT levy of 2.5%), which contributes over seventy percent of healthcare financing in Ghana [13, 14]. Since the NHIS inception over one and half decades ago, Ghana has made substantial progress in terms of coverage and improved.

health service utilization and health outcomes [12, 14, 17]. Notwithstanding the positive outcomes, there have been financial challenges in terms of reimbursement of claims to healthcare facilities [14].

Claims reimbursement to health facilities continue to be a challenge affecting quality healthcare provision [12, 13]. This has been a matter of concern and continue to draw the attention of researchers and stakeholders of the NHIS as well as the health system [12, 13]. It is a major topic of debate by all stakeholders. While challenges with regards to quality of healthcare service has been explored from different perspectives within the Ghanaian context under the NHIS [12, 17–20], there is limited evidence on the alternative local methods for healthcare financing in Ghana to inform policy. This study aimed at exploring health managers views on alternative domestic and sustainable methods of healthcare financing to inform policy.

## Methods

### Study setting

The study was undertaken in four district hospitals (Holy family hospital, Kwahu government hospital, Atua government hospital and St. Martins De Porres hospital) in the Eastern Region of Ghana. They comprised of public/government and mission or Christian Council Association of Ghana hospitals.

### Data management

#### Data collection and participants

A qualitative study using in-depth interviews with 16 health facility managers from four health facilities. The participants comprised of medical directors, insurance claims officers, hospital administrators and accountants.

The study used purposive and convenience sampling methods, The health managers and health facilities were purposively and conveniently selected to participate in the study [21].Using this methods we approached health facilities which were easily accessible to the researchers were approached and potential and qualified health managers who consented to participate in the study after the aim was explained to them were recruited to participate. In-depth interview guide with semi-structured questionnaire were used. Participants were assured of anonymity and confidentiality of the information that will be collected from them. A written consent was obtained from participants prior to participation in the interview. The interviews were tape-recorded with the permission of participants which lasted between 45 and 60 minutes.

### Data analysis

The recorded interviews were transcribed verbatim and analysed thematically using Clarke and Braun’s framework [22].Transcribed interviews were first read through word by word and codes were derived from the participants’ statements. Similar codes were grouped together into one theme or broader themes which were reviewed severally by the researchers. For coherency, preliminary themes within a theme were developed to aid interpretation and extracts to support narratives and analysis.

## Results

### Views on health managers on claims reimbursement to health facilities

Health managers’ views were sought on claims’ reimbursement and its effects on healthcare provision in the health facilities. Almost all the managers interviewed in the four facilities were not satisfied with how claims were reimbursed. That those submitted claims took a long time to be paid by NHIA. Apart from the irregularity of the reimbursement, it does not often cover the total amount owed/submitted. Some of their views are expressed in the following statements:

“……….. the claims reimbursement from the NHIA has been erratic. The authority does not pay according to the plan of four weeks as stated in the Act………. Reimbursement from the authority takes several months to come to hit our account. The more worrying thing is that whenever payment is made, it does not cover all the months owed us. For now, they owe our facility over eight to ten months but if they are paying, it will cover only two or three months which is not the best for hospital management……if you go to all the regions, they will tell you the same story….. it is really affecting us in several ways……there is need for lasting solution to this problem” [Hospital Administrator].

“……….. the claims payments to health facilities continue to become worst as days go by……. as it is not regular, the payments also come in bits as part payments sometimes covering few months out of the lot owed to the facility. It’s really affecting the operations of health facilities ……… it is a general issue for the facilities in the country. It’s really affecting our ability to manage our facilities…….” (Hospital Accountant).

### Challenges of claims reimbursement to health facilities

Healthcare managers acknowledged that the erratic nature of reimbursement affected their ability to provide standard healthcare as expected by their clients. A medical director expressed his opinion on the delayed claims reimbursement.

“…… the financial challenges are affecting our ability to provide the care required by our clients. …our situation now is like a boxer whose hands are been tied behind him and expected to fight his opponent in the ring and win the fight………… which is impossible………………. So, our situation is the same thing…. how can we provide quality or standard healthcare to our clients with resources…….? This is not possible ………………. We try to do our best though……………” (Medical director).

The participants also reported that the lack of regular claims reimbursement affect adequate provision of healthcare services to insured clients. A manager said:

“The financial challenge we are facing is also affecting our clients seriously……. they do not get the standard of quality services required from us. Sometimes we tell them to pay for some of the services  from their pocket or make co-payments that is when there are in stock. If the drugs are not in stock, we tell them or purchase them from outside the facility. They are always not happy when they hear this …. But there is nothing one can do……………….so that is the current situation. It’s really affecting all of us” (Medical director).

Concerning the same issue, views of the health insurance claim managers were not different.

“*If the poor which the insurance was meant to protect are now made to pay for healthcare services……… then it is no more serving the purpose………. then this is the time for the insurance managers and the government to rethink on how to overcome these financial threats to healthcare delivery*”

(Claims manager).

## Health managers views on reasons for delayed claims reimbursement.

We explored health managers views on reasons accounting for the delay in reimbursement of claims to the health facilities. This is a statement from one of the managers:

The delay in payment is due to the inability of the NHIA to provide enough funds for payment as a result of government’s failure to advance funds to the NHIA (Hospital administrator).

The managers also attributed the delay to politics and management issues as reflected in the following statements:

“There is meddling of politics in the management of the scheme’s affairs too much….it is not the best for sustainability for the NHIS (Hospital administrator).

### Views on how to address financial challenges for claims reimbursement

We sought participants views on how the current financial challenges of the NHIA can be tackled. Some participants believe that the only way to overcome the current financial challenges faced by the NHIA is to explore alternative and sustainable sources of funding by the government through the levying of special taxes on our natural resources and existing tax sources. A manager suggested:

“It is possible for the government to impose some levies from the proceeds from our natural resources such as gold, cocoa, bauxite and oil and gas to support health care in the country. If all these potential sources are available and the government is facing challenges of raising funds to finance healthcare…. it is very difficult to comprehend. The government need to explore these sources urgently since the NHIS is in financial crisis” (Medical director).

“I think raising the proportion of the current VAT to finance healthcare will not  be a bad thing at all…… that source has been supportive to the government and also reliable though…..” (Hospital accountant).

Other participants have the view that the government could raise additional funds by taxing telecommunication sector services. A participant said:

“……the government can raise additional funds locally by imposing special taxes on mobile money transfers to augment healthcare funds….” (Insurance claims officer).

Other participants also think that funds could be raised from taxes on airtime. A hospital administrator indicated:

*“By the government by introducing airtime tax to raise revenue to finance healthcare as done in some countries. We can also do that in Ghana. This is easy and reliable way for the government to raise more funds to support healthcare provision in the country”*

Participants think that while putting financial policies in place, pragmatic financial measures also need to be put in place to ensure judicious use of the funds for their intended purposes. A participant reported:

“…………any official or individual who misapply the scheme’s funds should be fined and prosecuted to serve as a deterrent to others or else……… if we fail to do that …. we can continue to put in more funds ………… but the financial challenges will persist” (Hospital accountant).

## Discussion

Our findings indicate a long-standing claims debt of several months owed to the healthcare facilities by the NHIA. Delayed claims reimbursement of service providers remains a key concern by NHIS-accredited health facilities in Ghana [12–14, 19, 23]. This has compelled them to sometimes demand out-of-pocket or co-payments from clients to be able to deliver services needed to them. Health managers expressed frustration in delayed reimbursement of claims by the NHIS. Delayed reimbursement coupled with poor quality of service remain a critical challenge that have the potential of reversing to out-of-pocket payment system in Ghana. It could also lead to decreased stakeholders’ trust and confidence [12, 14, 23, 24]. Health managers think there is the need for the NHIA and Government of Ghana to find immediate and sustainable financial solution to the current crisis to ensure timeous release of payments. According to WHO [25], to achieve sustainable quality health services, health systems should be capable of raising adequate funds for equitable health service provision without exposure to undue financial hardship [26]. However, ensuring adequate funding for healthcare by governments especially in LMICs like Ghana has been a herculean task over the years [27].

To help in addressing the current healthcare reimbursement challenges by the  NHIA to health facilities, the managers suggested alternative methods and sustainable local sources of funding to include: the levying  of special taxes on natural resources such as crude oil and gas, gold, bauxite, cocoa and other sources such as  mobile money transfers, airtime and increasing the proportion of levies on the existing VAT. Many LMICs over the past decade have implemented innovative local methods such as levies on mobile phone use to raise funds to finance UHC [28]. In Gabon, the government imposed 10% levy on mobile phone use in 2008 to fund for national health insurance system to finance critical healthcare services especially for the poor population [29]. In 2018, the government of Uganda imposed 0.5% tax on the value of mobile phones and 1% on value of mobile money withdrawals to raise additional funds for the health sector [28]. Similarly, raising additional funds to finance healthcare, the Zimbabwean government imposed a 3% levy on mobile airtime data [28]. Also, in Kenya, exercise taxes were imposed on money transfer services, such as airtime and internet data to finance healthcare [30]. Despite the potential of raising domestic funds through the imposition of levies on mobile phone services by Ghana to support UHC, it is crucial to access who bears the burden from the cost of special levies on mobile communication and money transfer services in Ghana. This calls for a further research to help the government of Ghana to implement taxation policies  to  ensure equity for better health.

In this study, apart from imposition of taxes on mobile services, to finance healthcare, the participants also suggested imposition  of special taxes on incomes from natural resources such as oil and gas, gold, cocoa, bauxite. In LMICs, Papua New Guinea, to raise an additional funds for the health sector, imposed special taxes on proceeds from copper and gold mines [27]. Similarly, in Botswana, the government imposed special taxes on diamonds to finance healthcare [28]. Also, in Angola, to raise funds through local sources to finance healthcare, the the government imposed levies on earnings from the export of crude petroleum [28].

Currently, in Ghana a 2.5% national health insurance levy from VAT, meets between 70 and 75% of Ghana’s healthcare financing needs [31, 32]. Health managers suggested that raising the proportion of the current VAT rate could help bring in additional funds to finance healthcare in Ghana. In LMICs, countries such as South Africa [28], Costa Rica, Malawi, Indonesia and Thailand have financed their healthcare through levies from VAT on goods and services [27, 31].However, governments and healthcare policy makers in LMICs are cautioned to find ways to minimize the regressive impact of indirect taxes on poor consumers to make the method a feasible and sustainable source of revenue for financing healthcare [6]. Despite the potential of these sources, it is important for governments and health policy makers in Ghana and other LMICs who are working towards implementing innovative local methods using special levies on mobile communication services and natural resources to finance their UHC, to implement those that best suit their economies to ensure equity for better health.

To ensure the judicious use and allocation of the proceeds or funds to enhance health services delivery, the managers suggested putting in place adequate monitoring measures and also  the prosecution of people who mismanage or apply the funds. They also think de-coupling of the management of the NHIA from political interference will help in the smooth running of its affairs. These recommendations are confirmed by other studies in Ghana [13, 33] however, this finding was not the main focus of this paper.

## Study limitations

This study has some limitations which should be noted. The study setting involved four healthcare facility managers in one region in Ghana, and the recommendations did not capture the general views of all healthcare facility managers in Ghana. However, these categories of managers are senior public health administrators, and their ideas or views will not differ significantly from the rest of the other healthcare facility managers in Ghana. As the first study to capture healthcare managers views on potential sustainable domestic sources of funding in Ghana, the instrument may be refined to capture the views of other stakeholders from the health sector.

## Conclusions

The study provides important insights into potential innovative alternative domestic sources for raising additional funds to finance healthcare services in Ghana. Despite the potential of these sources, it is important for governments and health policy makers in Ghana and other LMICs who are working towards implementing innovative local methods using special levies on mobile communication services and natural resources to finance their UHC, to implement those that best suit their economies to ensure equity for better health.

## Data Availability

The datasets used and/or analysed during the current study are available from the corresponding author on request.
